# Overcoming Voltage Losses in Vanadium Redox Flow Batteries Using WO_3_ as a Positive Electrode

**DOI:** 10.1002/cctc.202201106

**Published:** 2022-11-16

**Authors:** Seyedabolfazl Mousavihashemi, Sebastián Murcia‐López, Miguel A. Rodriguez‐Olguin, Han Gardeniers, Teresa Andreu, Juan Ramon Morante, Arturo Susarrey Arce, Cristina Flox

**Affiliations:** ^1^ IREC, Catalonia Institute for Energy Research Jardins de les Dones de Negre 1 Sant Adriá de Besós 08930 Spain; ^2^ Aalto University School of Chemical Engineering Kemistintie 1 Espoo 02015 Finland; ^3^ Mesoscale Chemical Systems MESA+ Institute University of Twente PO. Box 217 Enschede AE 7500 The Netherlands; ^4^ Institut de Nanociència i Nanotecnologia (IN2UB) Universitat de Barcelona Martí i Franques, 1 08028 Barcelona Spain; ^5^ Facultat de Física Universitat de Barcelona C. Martí i Franqués, 1 08028 Barcelona Spain; ^6^ Institut de Ciencia de Materials de Barcelona CSIC Campus UAB Barcelona 08193 Spain

**Keywords:** Positive Electrode, Pulsed Laser Deposition, Vanadium Redox Flow Batteries, Voltage losses, WO_3_

## Abstract

Vanadium redox flow batteries (VRFBs) are appealing large‐scale energy storage systems due to their unique properties of independent energy/power design. The VRFBs stack design is crucial for technology deployment in power applications. Besides the design, the stack suffers from high voltage losses caused by the electrodes. The introduction of active sites into the electrode to facilitate the reaction kinetic is crucial in boosting the power rate of the VRFBs. Here, an O‐rich layer has been applied onto structured graphite felt (GF) by depositing WO_3_ to increase the oxygen species content. The oxygen species are the active site during the positive reaction (VO_2_^+^/VO^2+^) in VRFB. The increased electrocatalytic activity is demonstrated by the monoclinic (*m*)‐WO_3_/GF electrode that minimizes the voltage losses, yielding excellent performance results in terms of power density output and limiting current density (556 mWcm^−2^@800 mAcm^−2^). The results confirm that the *m*‐WO_3_/GF electrode is a promising electrode for high‐power in VRFBs, overcoming the performance‐limiting issues in a positive half‐reaction.

## Graphical abstract

**WO_3_ for Vanadium Redox Flow Batteries**: Monoclinic (*m*)‐WO_3_ is deposited during pulsed laser deposition (PLD) over graphitic felt electrodes (GF). *m*‐WO_3_/GF is applied as a positive electrode in vanadium redox flow batteries (VRFBs). *m*‐WO_3_/GF minimizes the voltage losses, yielding excellent performance results in terms of power density output and limiting current density (556 mWcm^−2^@800 mAcm^−2^). From a broad perspective, PLD can be used to produce large‐scale area *m*‐WO_3_/GF electrodes.

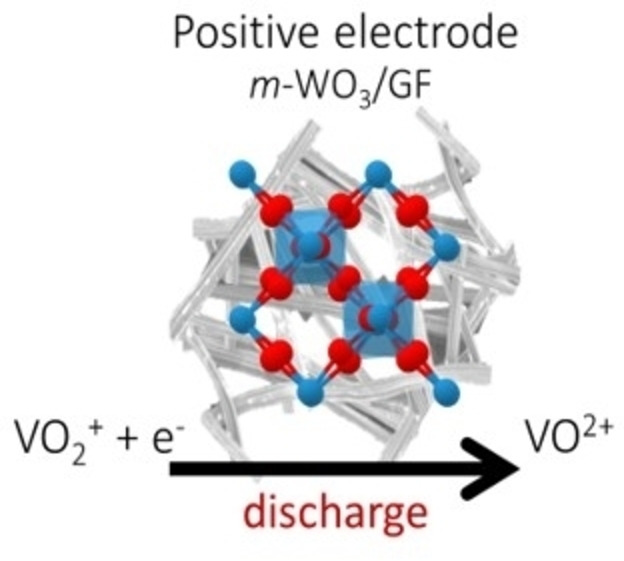

## Introduction

The need to provide clean and affordable energy that contributes to a sustainable future is becoming of paramount importance. Redox flow batteries (RFBs) are the most promising electricity‐storage system to address the intermittency issues of renewable energy sources, promoting smart‐grid applications and self‐consumption uses. In RFB technology, the active redox species are totally dissolved in the electrolyte solutions, which are stored in independent tanks and recirculated through the stack, where the redox reactions occur. The stack is the electrochemical converter based on the membrane, electrodes, and current collectors. Such as flow architecture promotes encouraging features in RFBs, such as decoupled energy and power output values, fast response time, and relatively easy scalability. The most developed and commercial RFB type is the vanadium redox flow batteries (VRFB), which exploits all oxidation states of vanadium (V^3+^/V^2+^ and VO^2+^/VO_2_^+^ for an anode and cathode, respectively. Since the same vanadium redox component is present in both compartments, the cross‐contamination issues are negligible, making this technology quite attractive. However, the practical application of VRFBs is limited due to the high capital investment cost. Besides the high cost related to the membrane and electrolyte, improved stack performance with a higher limiting current density and, consequently, a higher operational current density is desirable to reduce capital costs in VRFBs.^[1]^

Various reports have explored cell designs with novel flow fields achieving excellent results.^[2]^ Characteristics elements of the cell design are the separators and electrodes (cathode and anode). It is well known that the commercial electrodes present in VRFB cells have some shortcomings due to side reactions (*i. e*., hydrogen evolution reaction) and sluggish kinetic for a positive reaction, which induces voltage losses under operation, including activation and concentration polarization. Previous investigations demonstrated that the kinetics of the positive reaction (VO_2_^+^/VO^2+^) is slower than that of the negative electrode reaction (V^2+^/V^3+^), limiting the overall cell performance.^[3]^

Electrodes for the positive reaction can be modified to reach high efficiency and power density values. The oxygen species in O‐rich electrodes have been widely accepted as an active site for the oxidation of VO^2+^ to VO_2_^+^,^[2]^ with the concomitant electron transfer occurring via C−O−V bonds. Besides the mechanism activation for the O‐rich electrodes, the hydrophilic character of the GF can grant access to improve the contact volume of electrolyte, increasing interfacial electron transfer. In this regard, tungsten trioxide (WO_3_) is an O‐rich material that can promote electron transfer and be used for a positive oxidation reaction in VRFB. The WO_3_ has excellent stability in solid acid environments and can function as an electrocatalyst.[^4^, ^5^] WO_3_ can be deposited over GF via different simple and low‐cost methods, such as hydrothermal synthesis and impregnation.[^4^, ^6^] Depending on the synthetic route, WO_3_ could lead to a hexagonal, orthorhombic, triclinic, or orthogonal crystallographic phase (*h*‐ *o*‐, *t*‐, or *o_th_*‐WO_3_), which some of them could lead to enhancement in the electrochemistry of the VO_2_^+^/VO^2+^. Our previous work demonstrated that WO_3_ flower‐like nanostructures could be grown over the GF.^[7]^ The WO_3_ flower‐like nanostructures reveal an *h*‐WO_3_ phase after hydrothermal treatment steps. The steps are comprised of applying urea to functionalize the GF surface with N‐containing groups, followed by the addition of NaWO_3_ for introducing WO_3_ functionalities on the surface of GF with N‐containing groups. The approach enhances the power values and dramatically decreases the charge transfer resistance at the electrode/electrolyte interface. From the results, it can be argued that the crystalline phase plays a pivotal role. To date, the state‐of‐art contains successful *h*‐ or *o*‐WO_3_ phase functions in VRFB.[^6^, ^7^, ^8^, ^9^] However, little is known about *m*‐WO_3_ as a positive electrode in VRFB (Table S1).

The production of the *m*‐WO_3_ can be carried out using physical deposition approaches, such as pulsed laser deposition (PLD). The approach involves only one step to derive *m*‐WO_3_ over a planar or geometrical substrate.[^10^, ^11^, ^12^] The PLD offers several advantages over wet chemical methods.^[7]^ The most relevant is that PLD can lead to fewer WO_3_ crystallographic phase variations (Table S1). This is because PLD relies only on a target of defined crystallinity and composition (*e. g*., *m*‐WO_3_), which is then redeposited over a substrate.[^13^, ^14^, ^15^] Control over the WO_3_ crystallographic phase might offer superior performance during positive oxidation reactions in VRFB. For example, this could be the case for *m*‐WO_3_, which crystalline phase is considered more stable than the *h*‐WO_3_ at room temperature.[^16^, ^17^, ^18^] The main difference between *m*‐WO_3_ and *h*‐WO_3_ is the W and O arrangement within the WO_3_ crystallographic structure. The *m*‐WO_3_ contains O atoms bonded to W in a terminal position, while the *h*‐WO_3_ surface is formed with two kinds of O atoms; one type is adjacent to two O atoms, and another is adjacent to W and O atoms.^[19]^ This different arrangement in the oxygen structure results in much interest but is unexplored for *m*‐WO_3_ in VRFB.

In this work, we take advantage of the *m*‐WO_3_ derived from PLD. The *m*‐WO_3_ is deposited over complex geometries, such as GF. The deposition of *m*‐WO_3_ is confirmed with x‐ray diffraction (XRD). X‐ray photoelectron spectroscopy (XPS) confirms the presence of W^6+^ and O‐rich species, such as O^2−^. The O‐rich *m*‐WO_3_ contributes to increasing electrocatalytic properties within the VRFB. The results yield a massive drop in the voltage losses compared with the GF, overcoming the limitations of the positive half‐reaction in VRFB.

## Experimental section

The SGL Group kindly provided graphite felt (SIGRACELL, GF ∼2 mm‐thickness). In order to remove impurities, the graphite felt electrode was air‐treated at 400 °C for 30 hours in a tubular furnace. Hereafter, this electrode was labeled as GF and used for depositions of WO_3_ catalyst. The optimization of the deposition protocol has been reported elsewhere.^[11]^ In short, The WO_3_ thin‐film layer was deposited over the GF electrode by PLD in PLD‐5000 equipment (PVD products Inc.) with a 240 nm excimer KrF laser. A commercial target (American Elements Inc.) was used as a WO_3_ source. The distance between the target and the substrate was kept at 90 mm for all depositions, and the frequency and energy of the laser were set at 10 Hz and 140 mJ, respectively, while the temperature and oxygen pressure inside the chamber was 400 °C and 0.133 kPa. The number of pulses was used as a control parameter to modify and optimize the thickness of the films (ca. ∼150 nm). Hereafter, the WO_3_ GF electrode has been labeled as *m*‐WO_3_/GF electrode. All results are compared to the GF electrode as a control. In order to compare the electrochemical properties of both phases, *h*‐ and *m*‐WO_3_, the electrode *h*‐WO_3_/GF has been prepared using the same hydrothermal protocol as in our previous work.^[1]^

The WO_3_/GF electrode morphology was analyzed using a field emission (FE)‐scanning electron microscopy (SEM) using an FEI model Quanta FEG 650 instrument set at an accelerating voltage of 5 kV. Energy dispersive X‐ray spectroscopy (EDX) equipped with the SEM was used to obtain the elemental contents. The crystalline structure was analyzed with X‐ray diffraction (XRD) using a Bruker D8 Advance diffractometer with Cu Kα (1.54051 Å) radiation. X‐ray photoelectron spectroscopy (XPS) analysis was carried out using a PHI instrument model 5773 Multi‐technique with A1 Kα radiation (1486.6 eV) to analyze the chemical composition of the surface of the electrodes. The electron binding energies were referenced to carbon C 1s at 284.8 eV. High‐resolution spectra were fitted using previously fitting protocols[^20^, ^21^] (Lorentzian‐Gaussian line fitting and Shirley type) to compensate for the background.

The electrochemical properties were evaluated using cyclic voltammetry (CV) and electrochemical impedance spectroscopy (EIS) in a conventional three‐electrode cell. For this purpose, all as‐prepared electrodes (diameter 6 mm) act as the working electrode, and large Pt mesh and Hg/Hg_2_SO_4_/Sat. K_2_SO_4_ (0.664 V vs. SHE) electrodes were used as counter and reference electrodes, respectively. The electrolyte was 20 mL of 0.05 M of VOSO_4_ and 1 M H_2_SO_4_ solution, which was previously deaerated. In order to compare all as‐prepared electrodes in electrocatalytic activity, CV was performed at 1 mVs^−1^. After that, CV in the scan rates ranging from 1 to 10 mVs^−1^ was evaluated. Finally, the electron transfer properties were studied by EIS, applying an alternating voltage of 10 mV over the frequency range of 100 kHz to 10 mHz at open circuit potential.

The flow battery performance was evaluated by galvanostatic charge/discharge experiments and power peak values using a 3 cm^2^‐homemade cell published in previous research.[^7^, ^22^] Figure S1a shows the flow cell device with a zero‐gap cell – or filter press cell – connected to the electrolyte tanks through the tubing. The anolyte and catholyte electrolytes are stored independently in tanks, being continuously recirculated by two pumps to the electrochemical reactor. Inside the reactor, the electrolyte changes the oxidation state through electrochemical reactions over the electrodes. A filter press scheme is shown in Figure S1b, consisting of two compartments (*i. e*., anolyte and catholyte), both separated by the membrane. Each compartment contains electrodes and current collectors, where a flow field is graved to enhance the electrolyte/electrode interface. In all cases, the as‐prepared electrodes were used as the positive electrode, and GF was used as a negative electrode. Nafion® NRE‐212 was used as a membrane, and 20 mL of a 1.6 M V(IV) in 3 M H_2_SO_4_ solution was used as an electrolyte in each compartment. The theoretical capacity of the electrolyte was 858 mAh or 21.45 AhL^−1^ (using the total volume of the electrolyte, *i. e*., 40 mL). The flow rate of pumping positive and negative electrolytes into the cell was 30 mLmin^−1^ in all experiments, while nitrogen was bubbled through the negative tank during the process. Discharging polarization curves and peak power values were obtained following previously described procedures.^[23]^ In this case, the fully charged battery was discharged at the specified current density of 0 to 3,000 mAcm^−2^ for 30 s, obtaining the output voltage. Finally, a rest period of 2 minutes to a steady state at OCP was applied. The power density curves were obtained from the product of output voltage and the corresponding current density. All electrochemical experiments were carried out using a Biologic® VMP‐3 multi‐channel potentiostat controlled by EC‐lab® software.

**Figure 1:**
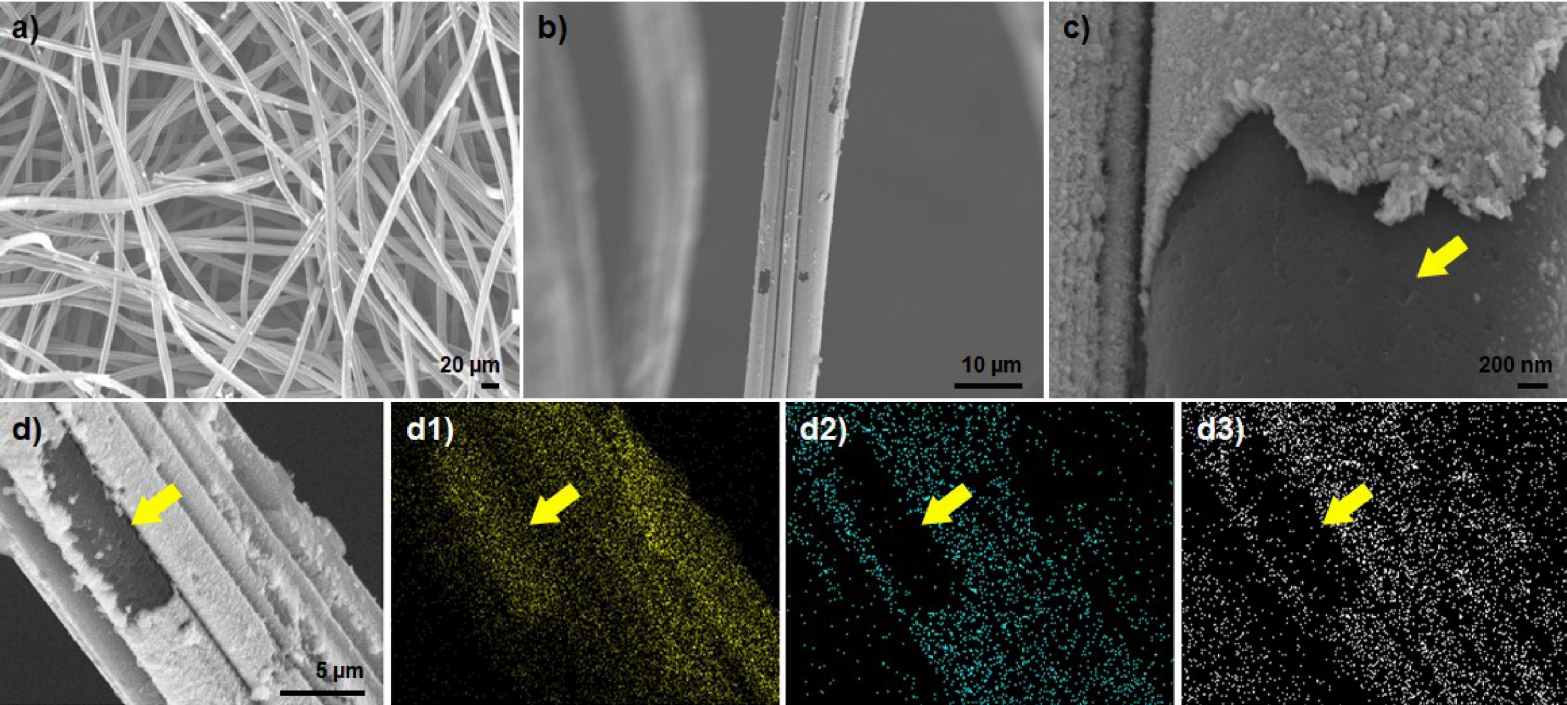
SEM image of (**a**) GF electrode; (**b–d**) *m*‐WO_3_/GF electrode. (**d**) SEM‐EDX mapping for the WO_3_/GF. The corresponding EDX maps are (**d1**) C signal in yellow, (**d2**) W signal in cyan, and (**d3**) O signal in gray.

## Results and discussions

The morphology of the produced *m*‐WO_3_/GF is shown in Figure 1. *m*‐WO_3_/GF electrode is constituted of several fibers stacked with fiber diameters of about 10–15 μm (Figure 1a), which can facilitate electrolyte transport during electrochemical reactions. An SEM image of a fiber bundle is also shown in Figure 1b. In general, *m*‐WO_3_ covering the fibers is observed, with minor uncoated areas corresponding to GF (dark gray). The presence of GF (yellow arrow) and the WO_3_ layer is confirmed with higher magnification images in Figure 1c. The WO_3_ layer exhibits column‐like crystal grains, densely aggregated and oriented perpendicular to the fiber surface (Figure 1c). The SEM estimated thickness of the WO_3_ layer is ∼150 nm. SEM‐EDX confirms the presence of C (yellow arrow), W, and O in Figure 1d. The results indicate a relatively good distribution of the *m*‐WO_3_ over the surface of the GC electrode.

**Figure 2:**
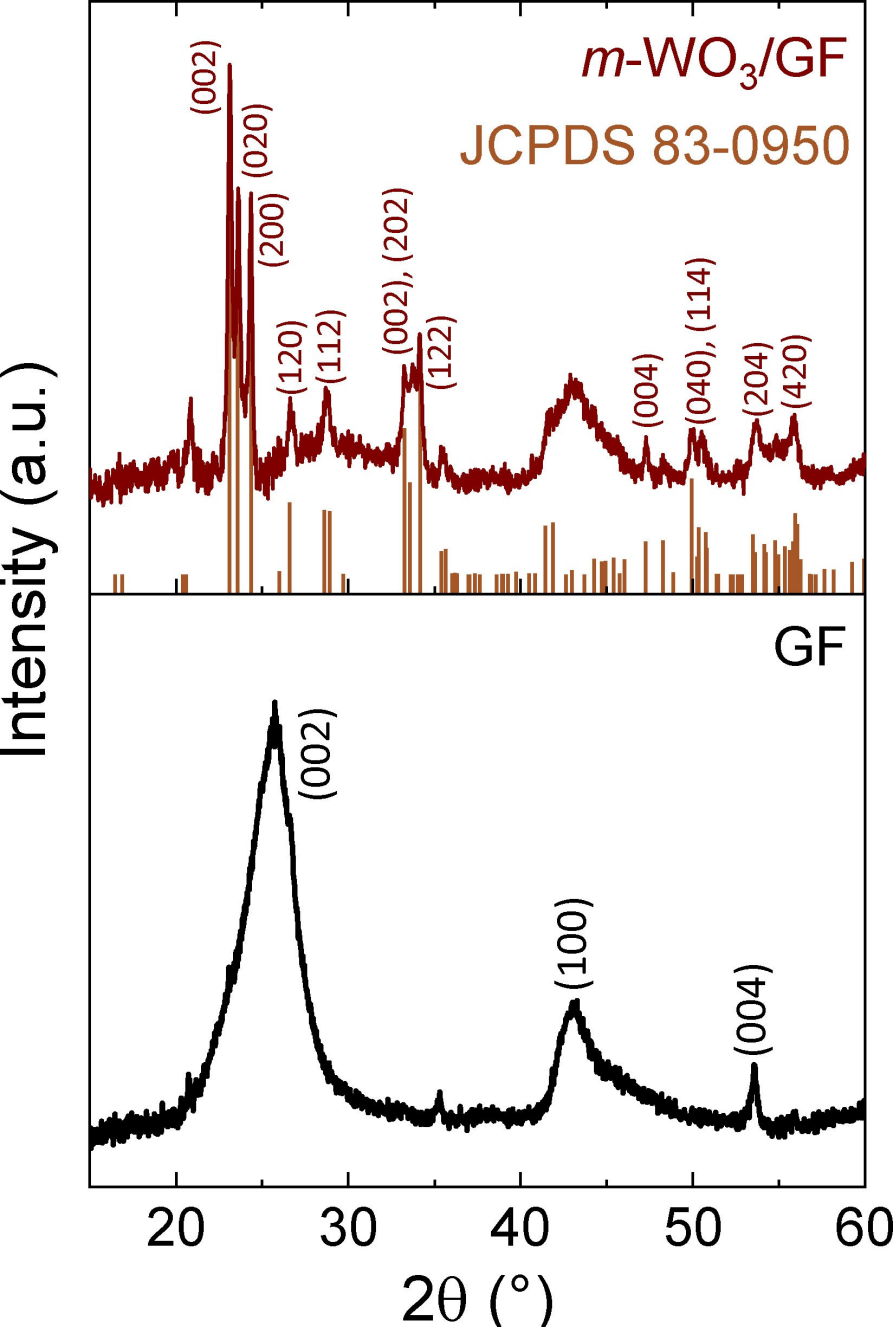
XRD pattern of the GF and *m*‐WO_3_/GF electrodes.

XRD diffraction for the GF and *m*‐WO_3_/GF electrodes is carried out to identify the WO_3_ crystallographic phase after the PLD process. The XRD in Figure 2 for the GF electrode reveals broad diffraction peaks around 2θ=24.45°, 43.4 °, and 53.5°, attributed to (002), (100), and (004) crystallographic planes of graphitic carbon electrode.[^7^, ^24^] In the same figure, the diffraction pattern for the *m*‐WO_3_/GF electrode matches with the JCPDS 83‐0950 card, in which the prominent peaks at 23.1°, 23.6°, and 24.4° are assigned to (002), (020) and (200) planes.[^25^, ^26^, ^27^] The GF XRD peak at 43.4° is observed in *m*‐WO_3_/GF XRD pattern. It is important to note that the GF XRD intense peak at 24.45° is not present in *m*‐WO_3_/GF. The absence of the GF XRD intense peak may be correlated to the high degree of crystallinity of the compact *m*‐WO_3_ layer over the GF. Previous work has found similar behavior, where the XRD pattern prevails for the crystalline *h*‐WO_3_ phase.^[7]^

**Figure 3:**
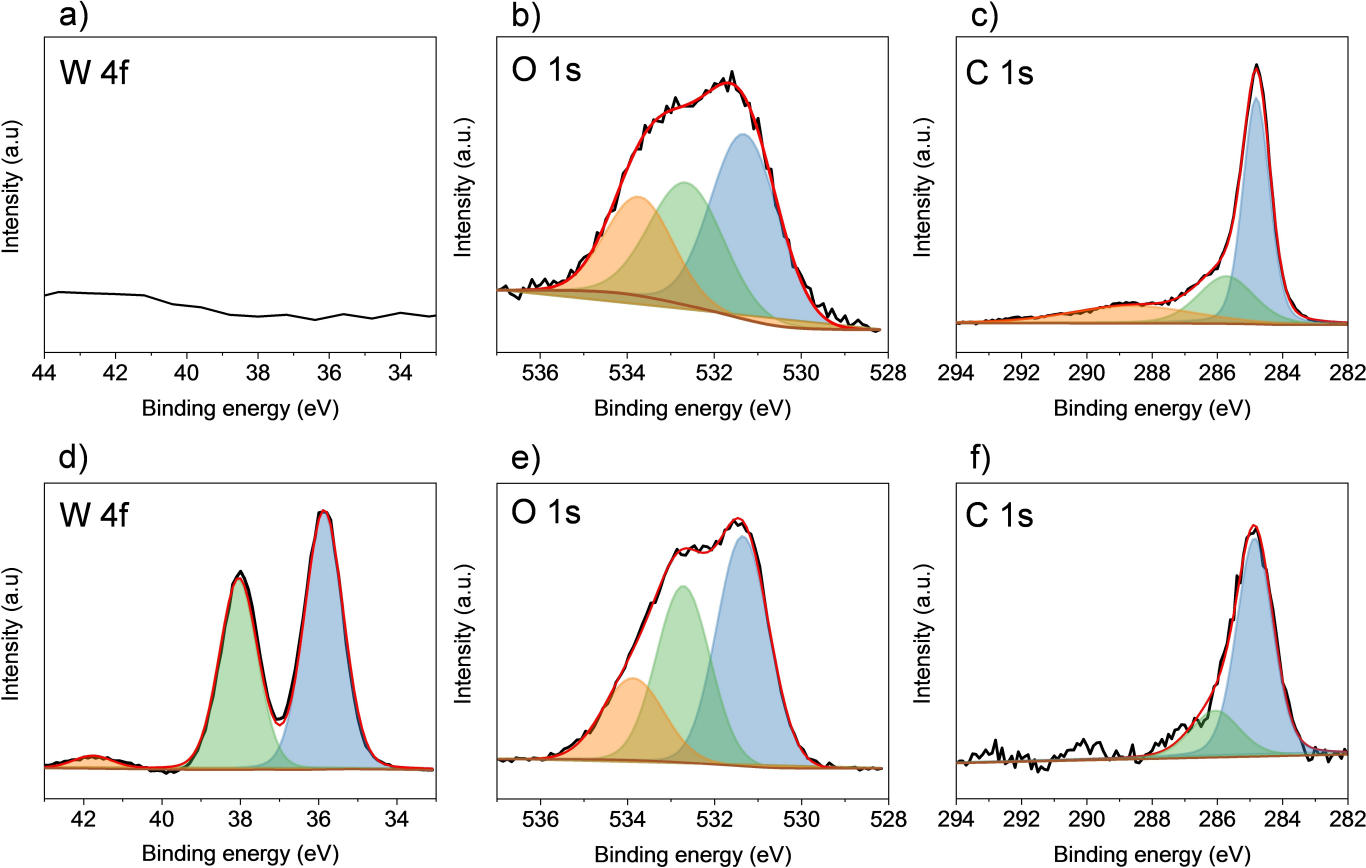
High‐resolution XPS for W 4f, O 1s, and C 1s score spectra for (**3 a**–**3 c**) GF and (**3 d**–**3 f**) *m*‐WO_3_/GF.

We turn to investigate the different species on the GF and *m*‐WO_3_/GF with XPS, comparing with the *h*‐WO_3_/GF. The high‐resolution XPS core spectra and the fitted curves for W 4f, O 1s, C 1s of GF, and *m*‐WO_3_/GF are shown in Figure 3. The GF does not show W (Figure 3a). As for the O 1s spectrum in Figure 3b, three peaks at 531.3, 532.6, and 533.7 eV have been fitted and attributed to C=O, C−OH, and C−C=O species.[^28^, ^29^, ^30^] In Figure 3c, the fitted peaks of the C 1s spectra for GF at 284.8, 285.7, and 288.6 correspond to C=C, C−C, and C=O species.[^28^, ^29^, ^30^] The chemical composition of GF is estimated using the general survey; in this case, 14.5 % of oxygen and 85.5 % of carbon have been measured (Figure S2 and Table S2).

High‐resolution core spectra for the *m*‐WO_3_/GF electrode are shown in Figure 3d–3f. In Figure 3d, the binding energies for W 4f_7/2_ at 35.8 eV and W 4f_5/2_ split‐orbit peak at 38.0 eV are attributed to W^6+^ in WO_3_.[^4^, ^5^, ^31^] The small peak at 41.7 eV corresponds to W 5p and is typically not used for the analysis. The O 1s high‐resolution core XPS for *m*‐WO_3_/GF in Figure 3e can be fitted into three peaks. The peak at 531.3 eV can be attributed to O_2_^−^ in the *m*‐WO_3_ lattice. The second peak at 532.7 eV is attributed to −OH species in WO_3_,[^32^, ^33^] which may overlap with the C−C=O species of the GF electrode. The third peak at 533.8 eV can be attributed to oxygen from adsorbed water on WO_3_[^31^, ^34^] with an additional overlap with the C−C=O species from the GF electrode. Although the overlaps exist, it is clear that oxygen content is higher when *m*‐WO_3_ is deposited over GF. . The attribution is due to a 5‐fold higher oxygen content (at. %) for *m*‐WO_3_/GF (Table S2). SEM and XRD further support the existence of the *m*‐WO_3_ layer in Figures 1 and 2. To further confirm our attributions on the type of O‐rich species presented over GF, *h*‐WO_3_ is synthesized following previously reported methodologies.^[7]^ XPS results from *h*‐WO_3_ in Figure S3 reveal a slight binding energy difference of −0.34 eV between *h*‐WO_3_ and *m*‐WO_3_ for W 4 f and O 1s, which could be attributed to the difference in the chemical environment of the samples, *e. g*., the presence of more reduced chemical species over the *m*‐WO_3_/GF. More importantly, the presence of oxygenated species for the *m*‐WO_3_/GF is more apparent than *h*‐WO_3_ (Figure S3). *h*‐WO_3_ species can be related to O_2_^−^ in the lattice and hydroxylated groups, in which OH^−^ adsorption can be favored by the presence of other species, such as defects.[^35^, ^36^] The results in Figure S3 indicate that *m*‐WO_3_ has more chemical species than *h*‐WO_3_, which could favor the vanadium redox process. Finally, the C 1s XPS spectrum for *m*‐WO_3_/GF in Figure 3f reveals the presence of two carbon species at 284.8 and 286.0 eV and are attributed to C=C, C−C.

**Figure 4:**
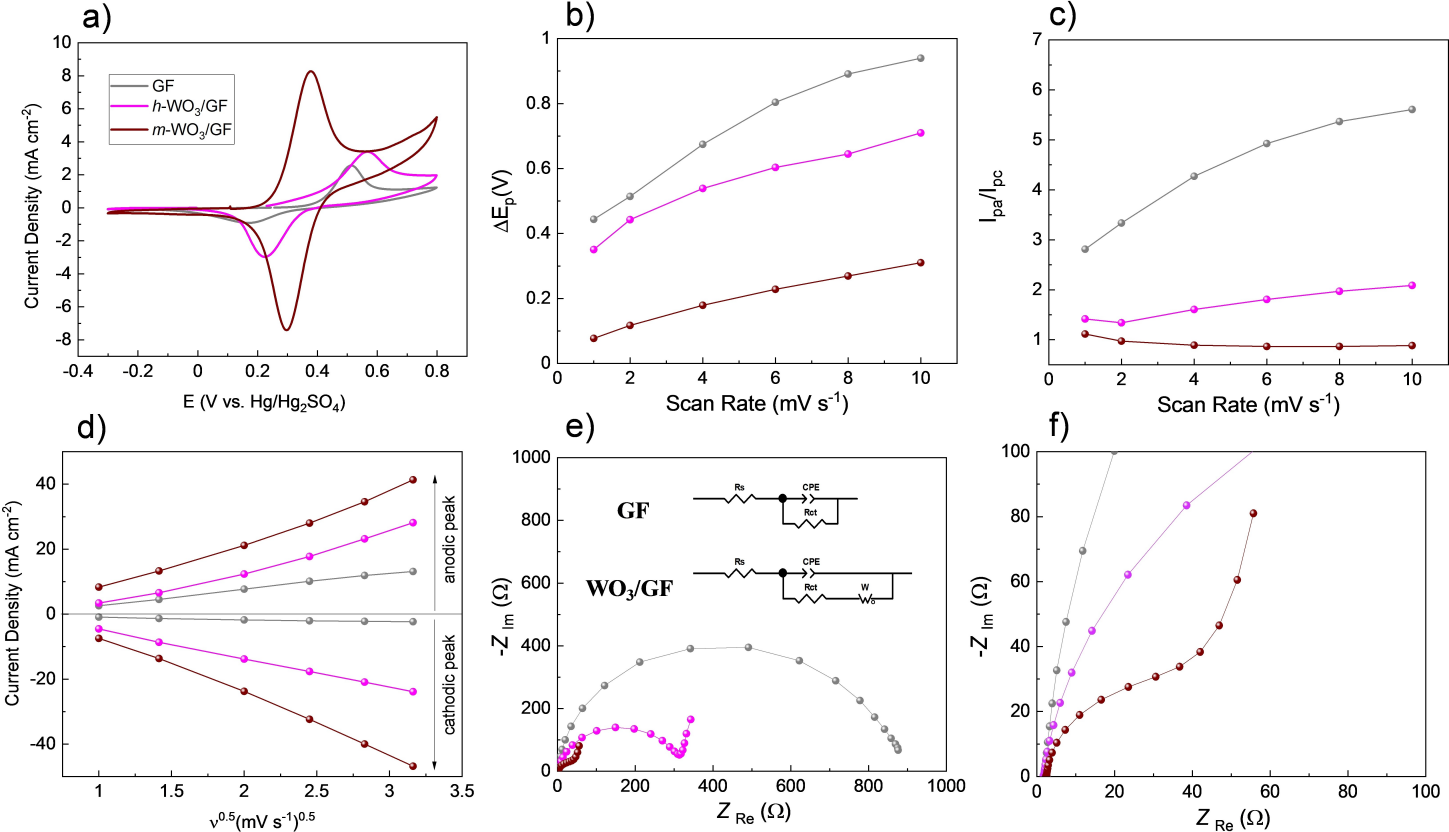
(**a**) Cyclic voltammetry results comparing GF (grey), *h*‐WO_3_/GF (pink), and *m*‐WO_3_/GF (magenta) electrodes at 1 mVs^−1^ scan rate. (**b**) peak‐to‐peak separation, and (**c**) *I*_pa_/*I*_pc_ ratio as a function of the scan rate. (**d**) The linear relationship between the peak current density and the square root of the scan rate. (**e–f**) Nyquist plot. Inset in (**e**): equivalent circuits used for modeling the experimental data.

The *m*‐WO_3_/GF functionality is demonstrated to be a promising catalyst for the VO_2_^+^/VO^2+^ redox reaction. It is generally accepted that the surface functional group of the electrode impacts the VO_2_^+^/VO^2+^ reaction kinetics. To corroborate this hypothesis for the case of the WO_3_/GF, CV and EIS experiments are carried out for all electrodes synthesized to elucidate their electrochemical properties toward positive half‐cell redox reactions in VRFB. Figure 4 shows the CVs profile comparison with and without WO_3_ towards the positive half‐cell reaction in VRFB at 1 mVs^−1^ scan rate in 0.05 M/1 M H_2_SO_4_ electrolyte solution (Figure 4a). The *m*‐WO_3_/GF electrode in Figure 4a displays the best electrocatalytic activity of all electrodes tested. In this case, the anodic and cathodic processes appeared as well‐defined peaks at 0.37 V and 0.30 V vs. Hg/HgSO_4_, respectively, exhibiting relatively good reversibility features (i. e., peak‐to‐peak separation less than 70 mV and *I*_pc_/*I*_pa_ closes to unity). Furthermore, the peak‐to‐peak separation (ΔE_p_) values increase with the scan rate for the *m*‐WO_3_/GF electrode (Figure 4b), while the I_pa_/I_pc_ ratio is closer to unity over the scan rate ranges studied (Figure 4c). An increase in the current density peak is also found, attaining values up to ca. 8.25 mA cm^−2^ (Figure 4a and Figure S4). Therefore, it is fair to say that the *m*‐WO_3_/GF electrode helps the kinetics of the positive reaction since potential and current density values are enhanced. In contrast to the results for *m*‐WO_3_/GF, the GF electrode exhibited lower peak potentials and current densities (Figure 4 and Figure S4), indicating poor activity. The latest behavior is also consistent with the scan rate from 1 to 10 mVs^−1^ (Figures 4b and 4 c), where an increment of ΔEp with the scan rate and I_pc_/I_pa_ values can be appreciated far away from the unity. CV results varying the scan rate for the synthesized *h*‐WO_3_ are plotted in Figure 4 and contrasted to all electrodes. As can be appreciated from Figure 4a and Figure S4, the *h*‐WO_3_ reveals an enhanced electrocatalytic activity compared with the unmodified GF electrode, demonstrating the important role of the O‐rich species. In fact, the oxidation and reduction peaks strongly appeared, but ΔE_p_ and the current densities for each process indicate poor electrocatalytic activity. Under the experimental conditions, the results show that *m*‐WO_3_ is a more active phase than *h*‐WO_3_, most probably due to the oxygenated species content (Figure S3). These results indicate that *m*‐WO_3_/GF has better reversibility for the positive reaction in VRFB.

The electrochemical surface area (ECSA) is investigated for *m*‐WO_3_/GF and GF to generate insights into the electrochemical surface area of the *m*‐WO_3_/GF electrodes. The ECSA is estimated using the Randles‐Sevcik equation (1) for a quasi‐reversible system and is presented in Figure 4c.

(1) $$I_{p}=2.99\;\times 10^{5}\;n\;{\left(\propto n\right)}^{1/2}A\;{D_{0}^{1/2}C}_{0\;}v^{1/2}$$

where *I*_p_ is the anodic peak current of oxidation peaks of VO_2_^+^ (A), *n* is the number of exchanged electrons, α is the transfer coefficient (0.5), *A* is the ECSA (cm^2^), *C_0_* is the initial concentration of the electroactive species (mol cm^−3^), *D_o_* is its diffusion coefficient (cm^2^ s^−1^), and *v* is the scan rate (V s^−1^). The values of the diffusion coefficient have been obtained from the literature.^[37]^

The slopes of the I_p_ vs. *v* ^1/2^ plots shown in Figure 4d allowed the calculation of the ECSA for each electrode. An increase in ECSA can be observed in the order (the ECSA value obtained in cm^2^ is given in parenthesis): GF (52.72)<*h*‐WO_3_/GF (120.50)<*m*‐WO_3_/GF (159.11). These results indicate that *m*‐WO_3_/GF electrode has more surface area available for electron transfer with more active sites in comparison with the GF electrode.

EIS experiments in Figures 4e–f are evaluated to understand the electron transfer properties of *m*‐WO_3_/GF and GF. The Nyquist plot obtained with *m*‐WO_3_/GF reveals two components: i) a straight line at low frequencies related to the diffusion of the ions and ii) a semi‐circle at high frequencies reflecting the electron transfer process. Therefore, the result demonstrates that the positive reaction presents charge transfer and diffusion‐controlled processes in the presence of *m*‐WO_3_. However, only the charge transfer process can be observed for GF the electrode. It is important to note that the GF electrode rendered inferior electron transfer properties when compared to *m*‐WO_3_/GF.

The equivalent circuit shown in the inset of Figure 4e simulates the classical Randles circuit for the aforementioned mixed control electrochemical process. The following elements can be appreciated in the equivalent circuit: 1) the Rs element represents the bulk electrolyte resistance; 2) R_ct_ denotes charge transfer resistance that occurred at the interface of the electrode material and the electrolyte; 3) the CPE is ascribed to a constant phase element, which is related to the double‐layer capacitance of the interface between the electrode and the electrolyte, and 4) the Warburg element is correlated to the diffusion of the vanadium ions through the electrolyte. The parameters obtained after the fitting data are listed in Table 1. The reported values are within the 10 % error or less.

**Table 1:** Parameters obtained from fitting the Nyquist plots with the equivalent circuit model

Sample	R_s_ [Ω]	R_ct_ [Ω]	CPE‐T [S.sec^n^]	CPE‐P	W‐R [Ω]	W‐T[S.sec^n^]	W‐P
GF	0.62	923.00	0.0003	0.95	–	–	–
*h*‐WO_3_/GF	1.75	301.60	0.0005	0.93	43.81	3.67	0.45
*m*‐WO_3_/GF	1.47	36.57	0.0130	0.98	60.02	4.92	0.44

Figure 4 and Table 1 results suggest that the enhanced electrochemical properties of the *m*‐WO_3_/GF electrode are caused by improved electron transfer properties and large active sites for the redox reaction. The results can be related to the amount of O‐rich species in *m*‐WO_3_ (Figure 3 and Figure S3). It is important to highlight the O species′ key role in the positive reaction mechanism. For example, compared with *h*‐WO_3_/GF electrode, the electron transfer resistance obtained was 301.6 Ω, while 36.57 Ω for the m‐WO_3_/GF electrode. The same trend can be appreciated for the CPE and W values, being the highest values of the CPE for the *m*‐WO_3_/GF electrode. This fact indicates that the *m*‐WO_3_ presents higher double capacitance of the electrode/solution interface, which can be ascribed to the O‐rich species that lead to superior electron transfer properties toward the VO_2_^+^/VO^2+^ reaction (Figure S4a).

**Figure 5:**
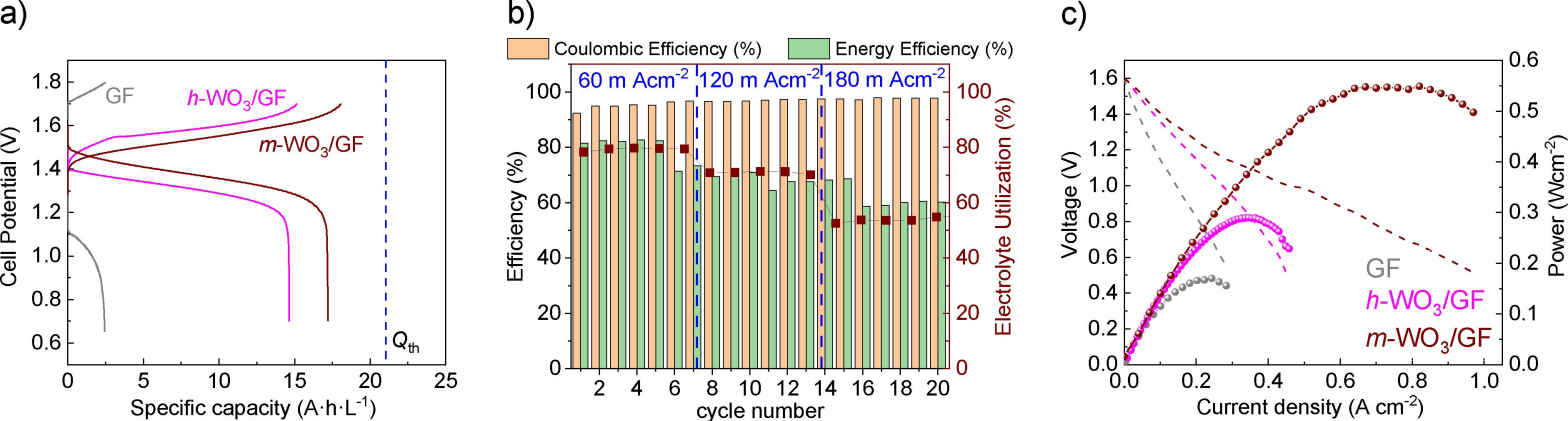
(**a**) Electrochemical performance GF, *h*‐WO_3_/GF, and *m‐*WO_3_/GF VRFB electrodes at 60 mA cm^−2^. (**b**) Evolution of the Coulombic capacity and energy efficiency versus the number of cycles at 60, 120, and 180 mA cm^−2^ for *m‐*WO_3_/GF in VRFB half‐reaction configuration. (**c**) Power density and polarization curves are for GF, *h*‐WO_3_/GF, and *m‐*WO_3_/GF positive electrodes tested within the full VRFB cell configuration.

Next, we paired the positive electrode (*i. e*., *h*‐WO_3_/GF, *m*‐WO_3_/GF, or GF) with a GF negative electrode to demonstrate the superior performance of *m*‐WO_3_/GF during VRFB. The VRFB full‐cell performance is measured under a galvanostatic charge/discharge experiment at several current densities to demonstrate the rate capability (Figures 5a and 5 b). The battery assembled with GF and *h*‐WO_3_ as positive electrodes shows poor performance, delivering 2.5 and 14.58 Ah L^−1^ at 60 mA cm^−2^, respectively. Conversely, the *m‐*WO_3_/GF electrode battery exhibits a remarkedly high capacity, attaining values up to 17.5 Ah L^−1^, which is close to the electrolyte theoretical capacity of the ca. 21 Ah L^−1^. The significant increment of the capacity is attributed to the *m*‐WO_3_‐layer catalyst, which contains O‐rich sites and enhances the positive half‐reaction at low charge transfer resistance.

We then investigate the VRFB Coulombic and Energy Efficienciesfor *m‐*WO_3_/GF and *h‐*WO_3_/GF in Figure 5b and Figure S4b. Indeed, the Coulombic efficiency denotes an excellent rate capability and stability of the *m‐*WO_3_/GF. Particularly, the Energy and the Coulombic efficiencies for *m‐*WO_3_/GF are ca. 82 % and 98 %, respectively, which remain stable for several cycles at 60 mA cm^−2^. As the current density applied increases, the energy efficiency and the full capacity tend to decrease, attaining values up to 70 % and 16 Ah L^−1^, respectively. For higher current densities (180 mA cm^−2^), the utilization rate drops to 56 %. The VRFB performance using *m*‐WO_3_/GF is improved regardless of the applied current density. Note that the utilization rate of the GF and *h*‐WO_3_‐positive electrodes is 10 % and 70 % at 60 m Acm^−2^. Particularly, the VRFB operating with an *h*‐WO_3_ catalyst dramatically decrease the performance when the high current density is applied (*i. e*., 180 mA cm^−2^), as shown in Figure S4b.

The improvements found in Figures 5a and 5 b resulted in the reduction of the electrochemical polarization of the positive reaction, effectively enhancing the positive half‐reaction in VRFB. Furthermore, the polarization curves (Figure 5c) obtained from the VRFB using *m*‐WO_3_/GF show that the activation, ohmic, and diffusion losses are reduced compared to the GF and *h*‐WO_3_‐positive electrodes. Considering that all VRFB are assembled with the same components except the GF, *h*‐WO_3_/GF, and *m‐*WO_3_/GF positive electrode, we can confirm that the O‐rich *m*‐WO_3_/GF electrode is responsible for the enhanced interfacial electron transfer and the reduced voltage losses in the positive reaction. Hence, *m*‐WO_3_/GF electrode can deliver a limiting current density of *ca*. 800 mA cm^−2^ with a power density peak of 556 mW cm^−2^, representing the highest value reported in the literature (Table S1).

## Conclusions

For the first time, an *m*‐WO_3_ layer with oxygen species deposited over GF has been applied in VRFB. The O‐rich layer composed of *m*‐WO_3_ has been obtained using PLD as a strategy to enhance interfacial electron transfer. The *m*‐WO_3_ interface can promote oxygen transfer processes, increasing the electrocatalytic properties of the positive reaction in VRFB. In particular, the *m*‐WO_3_/GF electrode decreases the electron transfer resistance from 923 Ω to 36.5 Ω. Such an improvement in the interfacial electron transfer impacts the kinetics of the positive reaction, making it feasible for the following key performance indicators: (**1**) ability to operate at 180 mA cm^−2^ with electrolyte utilization ratio of 56 % and energy efficiency of 70 %; (**2**) overcoming voltage losses related with the activation, ohmic and mass transfer, delivering a 556 mW cm^−2^ as power output and an 800 mA cm^−2^ as a limiting current density. These features open the door toward a new material implementation on the VRFB stack.

## Conflict of interest

The authors declare no conflict of interest.

1

## Supporting information

As a service to our authors and readers, this journal provides supporting information supplied by the authors. Such materials are peer reviewed and may be re‐organized for online delivery, but are not copy‐edited or typeset. Technical support issues arising from supporting information (other than missing files) should be addressed to the authors.

Supporting Information

## Data Availability

The data that support the findings of this study are available from the corresponding author upon reasonable request.
