# Supplementary material for: Overcoming Voltage Losses in Vanadium Redox Flow Batteries Using WO3 as a Positive Electrode
Source: ChemCatChem. 2022 Nov 16;14(23):e202201106. doi: 10.1002/cctc.202201106 (PMC10100004; doi:10.1002/cctc.202201106)
Supplement: Supplementary file 1 — Supporting Information [file CCTC-14-0-s001.pdf]

ChemCatChem

Supporting Information

Overcoming Voltage Losses in Vanadium Redox Flow Batteries Using WO_3 as a Positive Electrode

Seyedabolfazl Mousavihashemi, Sebastián Murcia-López, Miguel A. Rodriguez-Olguin, Han Gardeniers, Teresa Andreu, Juan Ramon Morante, Arturo Susarrey Arce,* and Cristina Flox*

Table of content

**Table S1.** Comparison of the power-related VRFB performance for GF and *m*-WO_3/GF	2
**Figure S1**. (**a**) Experimental flow cell device with the electrochemical reactor, electrolyte tanks and pumps; (**b**) Scheme of the electrochemical reactor.	5
**Figure S2**. General survey XPS for (a) GF and (b) WO_3/GF electrodes.	6
**Table S2**. XPS elemental composition (%at).	6
**Figure S3**. High-resolution XPS for W4f, O1s, and C1s score spectra for (3a-3c) PLD deposited *m*-WO_3/GF and hydrothermally deposited (3d-3f) *h*-WO_3/GF.	7
**Figure S4**. (**a**) Cyclic voltammetry study over several scan rates for GF, *h*-WO_3, and *m*-WO_3 electrodes. (**b**) Rate capability for VRFB using *h*-WO_3 catalyst as a positive electrode	8
**References**	9

Table S1. Comparison of the power VRFB performance for WO_3, GF and W-based electrodes

	**Approach**	**Crystalline phase**	**Electrolyte**	**Q/ (mL/min^{1})**	**J (mA/cm^{2})**	**CE/%**	**VE/%**	**EE/%**	**Power density (mW/cm^{2})**	**Limit current density (mA/cm^{2})**	**Reference**
m-WO_3/GF	PLD	Monoclinic	1.6M $VOSO_4$+ 3 M H_2SO_4	20	60	98	83.6	82	556	800	This work
o-WO_3/RDE	PLD	Orthorrombic	0.1 M VO^{2+} + 3 M H2SO4	NR	NR	NR	NR	NR	NR	NR	[1]
h-WO_3/CF	Hydrothermal	Hexagonal	1.8M $VOSO_4$+ 3 M H_2SO_4	20	200	96.3	67.9	65.4	350	400	[2]
h-WO_3/GF	Hydrothermal	Hexagonal	1 M VOSO4 + 3 M H2SO4	30L/h	70	99	78.9	78.1	NR	NR	[3]
					80	99.9	76.7	76.6			
					90	99.5	75.1	74.7			
					100	99.8	72.2	72.0			
h-WO_3/GF	Hydrothermal	Hexagonal	1.6 M VOSO4 in a 2.5 M H2SO4	30	40	90.7	92.3	83.7	NR	NR	[4]
					80	95.0	83.7	79.5			
					120	96.2	75.9	73.0			
					160	98.2	68.8	67.6			
Nb:*h*-WO_3	Hydrothermal	Hexagonal		30	40	92.4	92.1	85.1	NR	NR	[5]

			1.6 M $VOSO_4$ + 2.5 M H_2SO_4		80	93.2	83.8	78.1			
					120	95.1	75.0	71.3			
					160	95.3	69.1	65.8			
o-WO_3/C	Impregnation and oxidation	Orthorhombic	1.5 M V(IV) + 3 M H2SO4 and 1.5 M V(III) + 3 M H2SO4	NR	50	94.5	85.2	80.5	NR	NR	[6]
t-WO_3	Impregnation, annealing in air	Tetragonal	1.5 M $VOSO_4$ + 2 M H_2SO_4	Manual refill	80	90.2[a]	82	74	NR	NR	[7]
CF	-	NR	1M $VOSO_4$+ 3 M H_2SO_4	120	NR	NR	NR	NR	552	600	[8]
CF	-	NR	1M Vanadium + 5 M SO_4^{2-}	20	NR	NR	NR	NR	447	783	[9]
m-$W_{18}O_{49}$NW/GF	Hydrothermal, H_2 treatment	Monoclinic	1.6 M $VOSO_4$ + 3 M H_2SO_4	0.5 L/h	80	94.2	85.0	80.1	NR	NR	[10]
m-$W_{18}O_{49}$NW/GF	Hydrothermal, annealing in air	Monoclinic	1.6 M $VOSO_4$ + 3 M H_2SO_4	0.5 L/h	80	93.7	75.8	71.0	NR	NR	[10]

N:*h*-WC	Hydrothermal	Hexagonal	1.6 M VOSO4 + 3 M H2SO4	0.5L/h	100	94.91	78.86	74.85	NR	NR	[11]
c-WC/CNF	Electrospinning	Cubic	0.8 M VO^{2+} + 0.8 M V^{3+}+ 3.0 M H_2SO_4	NR	150	97.5[a]	70	68.3	NR	NR	[12]
c-WON	Impregnation, annealing in ammonia	Cubic	1.5 M $VOSO_4$ + 2 M H_2SO_4	Manual refill	80	91.0[a]	89	81	NR	NR	[7]

5

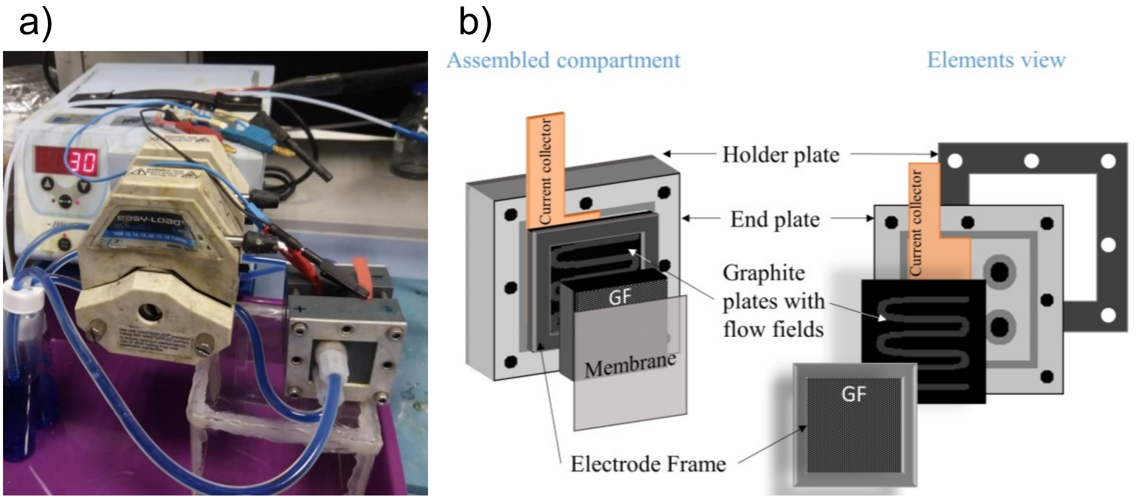

Figure 1. (a) Experimental flow cell device with the electrochemical reactor, electrolyte tanks, and pumps; (b) Scheme of the electrochemical reactor.

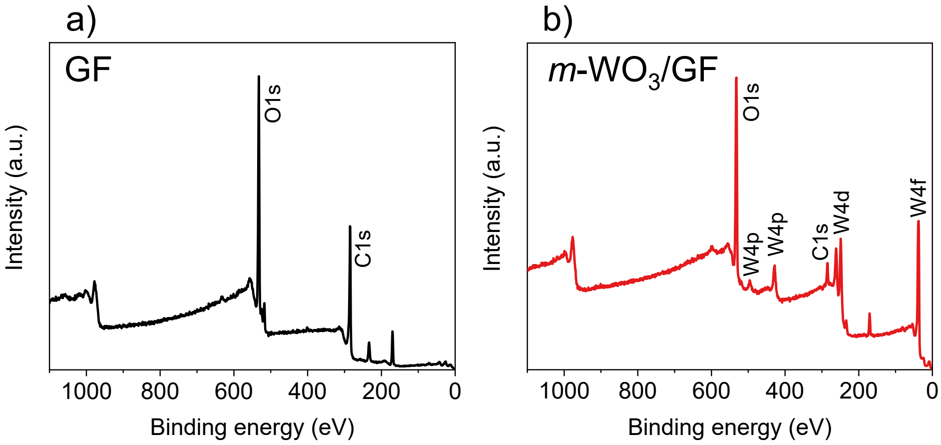

Figure S2. General survey XPS for (a) GF and (b) *m*-WO_3/GF electrodes.

Table S2. XPS elemental composition (% at).

Sample	W4f (%at)	O1s (%at)	C1s (%at)	**O/W ratio**
***m*-WO_3/GF**	13.89	73.23	12.88	5.27
**GF**	-	14.5	85.5	

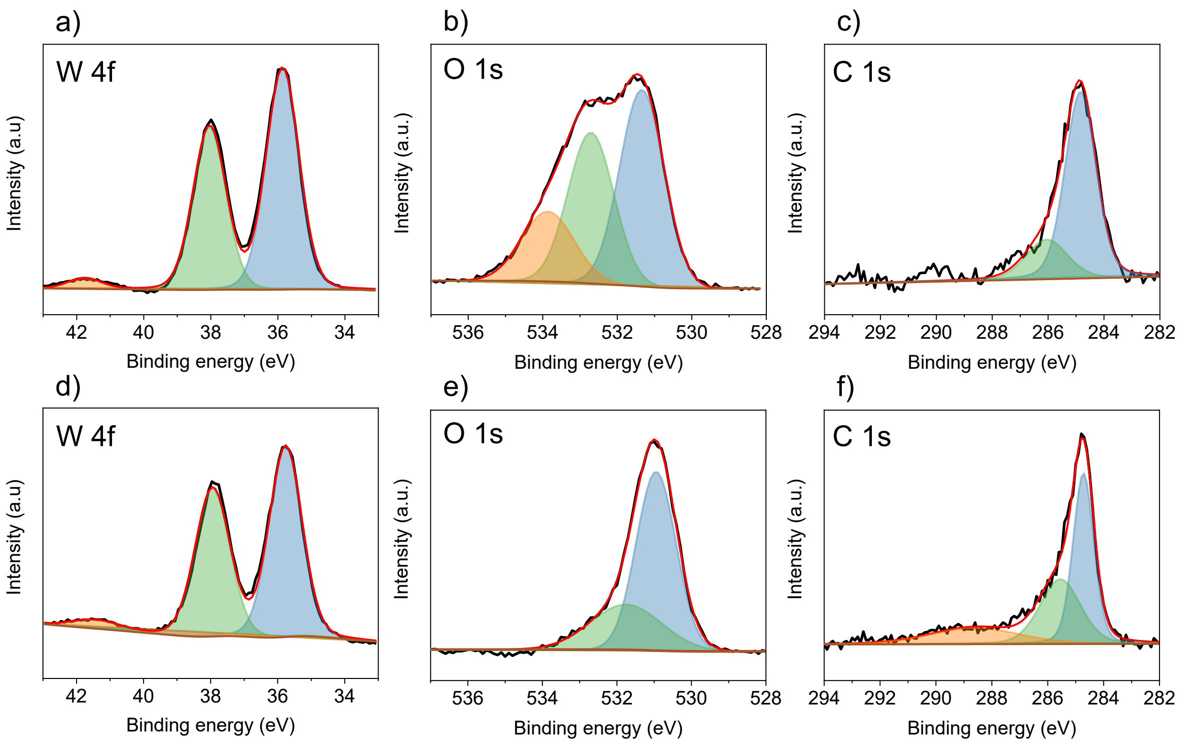

Figure S3. High-resolution XPS for W 4f, O 1s, and C 1s score spectra for (3a-3c) PLD deposited *m*-WO_3/GF and hydrothermally deposited (3d-3f) *h*-WO_3/GF.

A comparison between the high-resolution core spectra for *m*-WO_3/GF electrode and the *h*-WO_3 is shown in **Figure S2**. The W 4f, O 1s, C 1s high-resolution XPS core spectra and the fitted curves for *m*-WO_3 and *h*-WO_3 are presented in **Figures S2a-S2c** and **Figures S2d-S2f**, respectively. The results for **Figures S2a-S2c** are discussed in the main manuscript. In **Figure S2d**, the binding energies for W $4f_{7/2}$ at 35.7 eV and W $4f_{5/2}$ split-orbit peak at 37.9 eV are attributed to W^{6+} in WO_3.[4,5,13] The small peak at 41.4 eV corresponds to W 5p and is typically not used for the analysis. The O 1s high-resolution XPS core spectra for *h*-WO_3/GF in **Figure S2e** can be fitted into two peaks at 530.9 eV and 531.7 eV, which are attributed to O^{2-} in the WO_3 lattice and to -OH species in WO_3, respectively. [14,15] In **Figure S2f**, the fitted peaks of the C 1s spectra for *h*-WO_3 at 284.8, 285.5, and 288.6 correspond to C=C, C-C, and C=O species.[16–19]

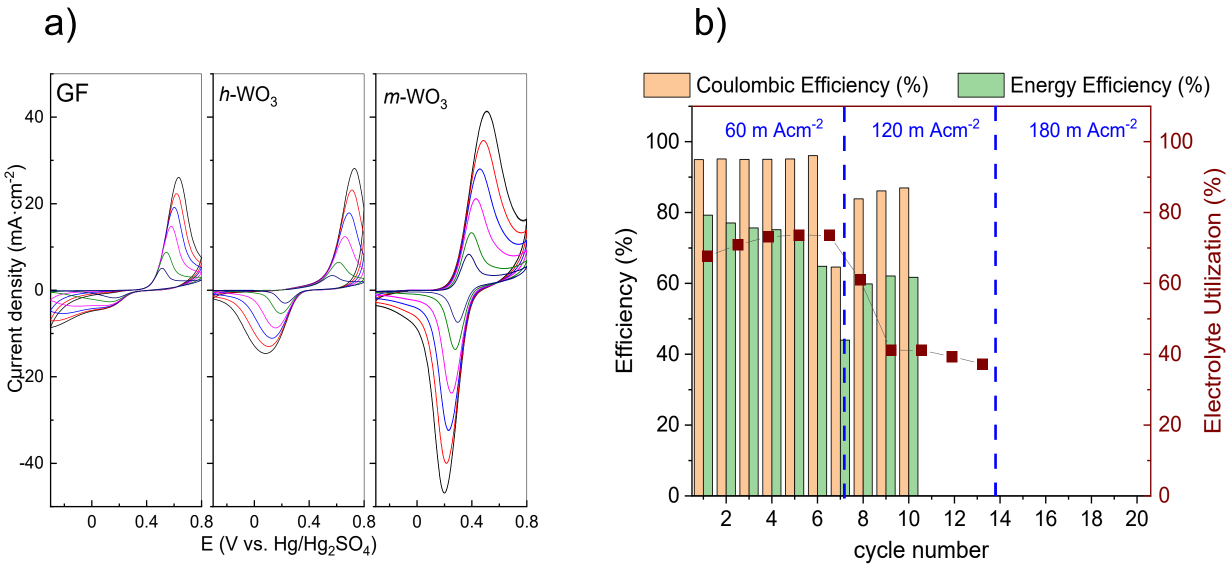

Figure S4. (**a**) Cyclic voltammetry study over several scan rates for GF, *h*-WO_3, and *m*-WO_3 electrodes. (**b**) Rate capability for VRFB using *h*-WO_3 catalyst as a positive electrode

References

[1] K. Kimura, Y. Miyahara, Y. Kondo, Y. Yokoyama, T. Abe, K. Miyazaki, *ChemElectroChem* **2021**, *8*, 3695.

[2] M. G. Hosseini, S. Mousavihashemi, S. Murcia-López, C. Flox, T. Andreu, J. R. Morante, *Carbon N Y* **2018**, *136*, 444.

[3] Y. Shen, H. Xu, P. Xu, X. Wu, Y. Dong, L. Lu, *Electrochim. Acta* **2014**, *132*, 37.

[4] D. M. Kabtamu, Y. C. Chang, G. Y. Lin, A. W. Bayeh, J. Y. Chen, T. H. Wondimu, C. H. Wang, *Sustain Energy Fuels* **2017**, *1*, 2091.

[5] D. M. Kabtamu, J. Y. Chen, Y. C. Chang, C. H. Wang, *J. Mater. Chem. A Mater* **2016**, *4*, 11472.

[6] C. Yao, H. Zhang, T. Liu, X. Li, Z. Liu, *J Power Sources* **2012**, *218*, 455.

[7] W. Lee, C. Jo, S. Youk, H. Y. Shin, J. Lee, Y. Chung, Y. Kwon, *Appl. Surf. Sci.* **2018**, *429*, 187.

[8] H. Sharma, M. Kumar, *J. Power. Sources* **2021**, *494*, 229753.

[9] I. Mayrhuber, C. R. Dennison, V. Kalra, E. C. Kumbur, *J. Power. Sources* **2014**, *260*, 251.

[10] A. W. Bayeh, D. M. Kabtamu, Y. C. Chang, G. C. Chen, H. Y. Chen, T. R. Liu, T. H. Wondimu, K. C. Wang, C. H. Wang, *ACS Appl. Energy Mater* **2019**, *2*, 2541.

[11] A. W. Bayeh, D. M. Kabtamu, Y. T. Ou, N. Y. Hsu, H. H. Ku, Y. M. Wang, T. C. Chiang, H. C. Huang, C. H. Wang, *ACS Sustain. Chem. Eng.* **2022**, 10, 12271

[12] G. Cheng, Y. Jiang, Y. Li, J. Chen, Z. He, W. Meng, L. Dai, L. Wang, *Electrochim. Acta* **2020**, *362*, 137178.

[13] Z. Na, X. Wang, D. Yin, L. Wang, *Nanoscale* **2018**, *10*, 10705.

[14] H. Idriss, *Surf. Sci.* **2021**, *712*, 121894.

[15] Y. Liu, J. Li, H. Tang, W. Li, Y. Yang, Y. Li, Q. Chen, *Electrochem. Commun.* **2016**, *68*, 81.

[16] D. M. Kabtamu, J. Y. Chen, Y. C. Chang, C. H. Wang, *J. Power Sources* **2017**, *341*, 270.

[17] Y. C. Chang, J. Y. Chen, D. M. Kabtamu, G. Y. Lin, N. Y. Hsu, Y. S. Chou, H. J. Wei, C. H. Wang, *J. Power Sources* **2017**, *364*, 1.

[18] W. A. Daoud, X. Xie, Y. Xiang, *ACS Appl. Energy Mater* **2020**, *3*, 10463.

[19] A. B. López-Oyama, R. A. Silva-Molina, J. Ruíz-García, R. Gámez-Corrales, R. A. Guirado-López, *J. Chem. Phys.* **2014**, *141*, 174703.
